# The Inflammatory Pattern of Chronic Limb-Threatening Ischemia in Muscles: The TNF-α Hypothesis

**DOI:** 10.3390/biomedicines10020489

**Published:** 2022-02-18

**Authors:** Diego Caicedo, Clara V. Alvarez, Sihara Perez-Romero, Jesús Devesa

**Affiliations:** 1 Angiology and Vascular Surgery Department, Complejo Hospitalario de Santiago de Compostela, 15706 Santiago de Compostela, Spain; 2 Neoplasia & Endocrine Differentiation P0L5, Centro de Investigación en Medicina Molecular y Enferme-dades Crónicas (CIMUS), University of Santiago de Compostela (USC), 15783 Santiago de Compostela, Spain; clara.alvarez@usc.es (C.V.A.); siara.perez@usc.es (S.P.-R.); 3 The Medical Center Foltra, 15886 Teo, Spain; devesa.jesus@gmail.com

**Keywords:** vascular inflammation, peripheral arterial disease (PAD), chronic limb-threatening ischemia (CLTI), GHAS trial, TNF-α, hsCRP, neutrophil-to-lymphocyte ratio (NLR), *NOX4*, *NOS3* (*eNOS*), *VEGFA*

## Abstract

**Background**: Vascular inflammation plays a crucial role in peripheral arterial disease (PAD), although the role of the mediators involved has not yet been properly defined. The aim of this work is to investigate gene expression and plasma biomarkers in chronic limb-threating ischemia (CLTI). **Methods:** Using patients from the GHAS trial, both blood and ischemic muscle samples were obtained to analyze plasma markers and mRNA expression, respectively. Statistical analysis was performed by using univariate (Spearman, t-Student, and X^2^) and multivariate (multiple logistic regression) tests. **Results:** A total of 35 patients were available at baseline (29 for mRNA expression). Baseline characteristics (mean): Age: 71.4 ± 12.4 years (79.4% male); TNF-α: 10.7 ± 4.9 pg/mL; hsCRP:1.6 ± 2.2 mg/dL; and neutrophil-to-lymphocyte ratio (NLR): 3.5 ± 2.8. Plasma TNF-α was found elevated (≥8.1) in 68.6% of patients, while high hsCRP (≥0.5) was found in 60.5%. Diabetic patients with a high level of inflammation showed significantly higher levels of NOX4 expression at baseline (*p* = 0.0346). Plasma TNF-α had a negative correlation with *NOS3* (*eNOS)* expression (−0.5, *p* = 0.015) and plasma hsCRP with *VEGFA* (−0.63, *p* = 0.005). The expression of *NOX4* was parallel to that of plasma TNF-α (0.305, *p* = 0.037), especially in DM. Cumulative mortality at 12 months was related to NLR ≥ 3 (*p* = 0.019) and TNF-α ≥ 8.1 (*p* = 0.048). The best cutoff point for NLR to predict mortality was 3.4. **Conclusions:** *NOX4* and TNF-α are crucial for the development and complications of lower limb ischemia, especially in DM. hsCRP could have a negative influence on angiogenesis too. NLR and TNF-α represent suitable markers of mortality in CLTI. These results are novel because they connect muscle gene expression and plasma information in patients with advanced PAD, deepening the search for new and accurate targets for this condition.

## 1. Introduction

Peripheral arterial disease (PAD) is an occlusive arterial disease, mainly of atherosclerotic origin, that affects lower limbs. It represents an independent risk factor for cardiovascular (CV) morbidity and mortality [1,2,3]. In fact, these patients have a CV event rate similar to those with established coronary or cerebral vascular disease [4,5,6]. Considering that there are few tools to identify which patients with PAD are at risk for an acute event, risk handling in this setting represents a major health challenge [4,7]. Inflammation plays a key role in the development of PAD, but the mediators involved in this condition have not yet been fully defined [8,9]. The isolated use of clinical staging of PAD (Rutherford class) ignores the importance of other factors that may also be crucial for long-term survival in these patients, such as biomarkers of systemic inflammation or endothelial dysfunction [9,10].

Both experimental and large cohort studies in humans have evaluated the role of inflammatory cytokines in atherosclerosis [3,11,12,13,14]. IL-6, TNF-α, and C-reactive protein of high sensitivity (hsCRP) have been found predictive of future CV events in healthy and diseased populations [15,16]. However, studies using Mendelian randomization have shown contradictory results depending on the marker, justifying the need for further study [9,15,17,18,19]. Moreover, it seems that different cytokines could influence the diverse vascular beds in different ways. In fact, murine and human data do not always coincide, as we will see below.

Inflammation has a clear deleterious effect on vessel endothelial function. Indeed, the CANTOS trial provided the first compelling evidence that inhibition of cytokine function, by decreasing the activity of IL-1ß and IL-6 signaling pathways, can reduce CV risk regardless of blood pressure or lipid level [20]. However, this breakthrough has only been demonstrated so far for coronary heart disease. In PAD, several cytokines have been associated with disease progression in one cohort study, finding a significant increase in the levels of IL-6, TNF-α, selectins, neopterin, CAMs, MMP-2, and MMP-9 [21]. However, a preceding study found no significant difference for IL-6 and IL-1ß in lower limb ischemia [22]. While in the CANTOS study the relevant role is for IL-6, in PAD the levels of TNF-α and IL-8 were clearly increased, which supports the statement of different inflammatory patterns in both atherosclerotic conditions. Further, IL-6 secretion is highly dependent on TNF-α and appears to have different behavior when secreted by monocytes (proinflammatory) or skeletal muscle (anti-inflammatory) [23]. In addition, nonrandomized observational studies have suggested a reduction in the rate of atherosclerotic events in patients treated with TNF-α inhibitors [24,25]. However, the causal association between these or other biomarkers and PAD has not yet been established.

The neutrophil-to-lymphocyte ratio (NLR), defined as the ratio between absolute count of neutrophils and lymphocytes, has been gaining relevance as a marker of CV disease. Many researchers have deeply evaluated NLR as a potential prognostic biomarker, predicting pathological and survival outcomes in patients with atherosclerosis and, in particular, in PAD, in which a strong relationship has been identified for systemic (long-term mortality) and local (lower limb) complications [26].

It is also well known that oxidative stress plays an important role in endothelial dysfunction in the inflammatory context of CV disease. Many studies have highlighted how oxidative distress triggers and impairs this condition, facilitating adverse CV events [27,28]. Although many studies currently address inflammation and redox balance in the vascular system, few of them have cross-linked gene and plasma information in a human trial.

Here we present new insights from an interventional study, the GHAS trial, in which information about muscle tissue mRNA expression and plasma biomarkers was combined in patients suffering from chronic limb-threatening ischemia (CLTI). Our results are novel because they were obtained in this PAD special subset of patients presenting CLTI, who represent those with the highest level of inflammation and CV risk.

## 2. Materials and Methods

From January 2016 to December 2018, all patients with diagnosis of CLTI who met inclusion criteria were enrolled in the Growth Hormone Angiogenic Study (GHAS) trial, registered in the Spanish Registry of Clinical Trials (REEC) with the number 2012-002228-34.

The GHAS study, a phase III randomized controlled trial, was designed to test the benefit of low dose of GH (0.4 mg per day, 5 days a week, during 2 months) for wound healing and rest pain relief in CLTI patients compared to placebo or control group, in which an injection of serum was administered with the same protocol instead of GH [28,29,30]. Blood samples were extracted for the determination of plasma biomarkers at the beginning of the study (basal) and after two months of treatment initiation (final). Skeletal muscle samples from the ischemic limb were also obtained with the same time interval.

### 2.1. Informed Consent and Recruitment

Written informed consent was obtained from each participant at the beginning of the investigation. The recruitment of the subjects for this study was made by vascular surgeons in the Angiology and Vascular Surgery Department of the Clinical Hospital of Pontevedra, Spain.

### 2.2. Medical Screening through Medical History and Physical Examination

At the beginning and at the end of this study, demographic information, CV risk factors, comorbid conditions, Rutherford class of ischemia, ankle–brachial index (ABI), ankle pressure (AK) and photoplethysmography (PPG), blood and muscle samples, and a list of current medications were collected from the medical history of each patient.

### 2.3. Inclusion/Exclusion/Withdrawal Criteria

#### 2.3.1. Inclusion Criteria:

– Age > 18 years
– Diagnosis of CLTI: presence of trophic lesions and/or rest pain plus ABI less than 0.4 and/or AP < 50 mmHg or plain or damped PPG curves or toe pressure (TP) < 30 mmHg [31].
– Failure of a previous attempt of revascularization; patients considered at high risk of failure or at high risk of surgical complications during the procedure or in poor condition for surgery.
– High risk of limb loss.

#### 2.3.2. Exclusion Criteria

– Pregnancy
– Legally incapacitated.
– Current cancer or during the last 5 years before the study.
– Current pneumonia or sepsis or severe foot infection.
– Untreated hypothyroidism and/or hypocortisolism.

#### 2.3.3. Withdrawal Criteria

– Patient’s own request.
– Decision of the physician due to adverse reactions supposedly secondary to the drug.
– Pneumonia/sepsis during the period of treatment.
– Increase in levels of IGF-1 more than 2 standard deviations.
– Increase in tumor markers.

A total of 37 consecutive subjects met the inclusion criteria. There were 2 deaths after signing the informed consent and before starting any treatment. Therefore, 35 patients were finally eligible for the study. However, both baseline and final muscle samples were obtained in 29 patients who completed the trial without amputation in the referred treatment period (2 months). A total of 16 patients were treated with GH (GH group), and 13 received placebo (control group or placebo group).

### 2.4. Measurements

#### 2.4.1. ABI and AP

The method used for determination of ABI and AP has been extensively described and recently reviewed [32,33]. A bidirectional continuous doppler with an 8 MHz probe and specific software (Hadeco, es-100V3 model, Quermed S.A, Madrid, Spain) was used for curve analysis.

#### 2.4.2. Inflammatory and Vascular Circulating Biomarkers

A blood sample from a peripheral vein was obtained for determination of plasma biomarkers. The markers and their reference values were: tumor necrosis factor-alpha (TNF-α): <8.1 pg/mL; high-sensitivity C-reactive protein (hsCRP): <0.5 mg/dL or 5mg/L; beta-2 microglobulin (ß-2M): <0.25 mg/L; cystatin C (CyC): 0.53–0.95 mg/L; fibrinogen: 200–430 mg/dL; glycosylated hemoglobin (HbA1C): <5.5%; insulin-like growth factor I (IGF-1) and IGF-1 binding protein 3 (IGF-1-BP3): age-standardized values according to our laboratory reference values. Quantifications were performed by using ELISA technique according to the manufacturer’s protocols. TNF-α, IGF-1, and IGF-BP3 were measured using IMMULITE 2000 IMMUNOASSAY SYSTEM (Siemens); for ß-2M and CyC the DIMENSION VISTA 1500 (Siemens) was used; for hsCRP, ADVIA 2400 CHEMISTRY SYSTEM (Siemens); for HbA1C, HPLC ADAMS A1C HA-8180 (Arkray); cell count was performed using ADVIA 2120 HEMATOLOGY SYSTEM (Siemens); fibrinogen was measured using ACL TOP 550 (Werfen).

The reference value of NLR in general population is considered 2.15. In PAD patients, it ranges between 2.5 and 5.25 in different studies. A cutoff point for elevated NLR has not been properly defined for PAD, though the recommendation is to consider a value between 2.5 and 3 [26]. In our study, a value ≥ 2.5 was chosen as elevated.

#### 2.4.3. Skeletal Muscle Samples

Samples were taken from the soleus muscle using a cutting trocar and local anesthesia with 2% lidocaine. In the internal aspect of the leg, a small skin incision of 2–3 mm was made. Then, the trocar was inserted until the desired level, and a muscle cylinder was removed. Once extracted, the samples were conserved in RNA-later (AM7021, Invitrogen, Vilnius, Lithuania) at 4 °C and finally stored at −80 °C until analysis.

#### 2.4.4. Real-Time PCR (RT-qPCR)

RNA extraction was performed using TRIzol™ reagent (15596026, Invitrogen, Carlsbad, CA, USA), following manufacturer’s instructions. RNA was incubated with 1 IU RNase free DNase I (EN0521, Thermo, Carlsbad, CA, USA), 5 μL 10X buffer with MgCl2, and water for a total volume of 50 μL at 37 °C for 30 min. The reaction was terminated by inactivating DNase, and then RNA was purified with an affinity column using the GeneJET RNA Cleanup and Concentration micro kit (K0842, Thermo Fisher, Vilnius, Lithuania). RNA was finally quantified by spectrophotometry (Nanodrop 2000, Thermo Fisher, Vilnius, Lithuania).

Total RNA pool obtained from commercial human skeletal muscle was used as reference (636534, Clontech, Mountain View, CA, USA).

A total of 1 µg of total, previous to cDNA synthesis, is incubation with 1 IU of RNase-free DNase I (EN0521, Thermo Fisher, Carlsbad, CA, USA), 1 μL of MgCl2 buffer, and water to a final volume of 10 μL for 30 min at 37 °C. DNase was then inactivated by adding 1 μL of EDTA and incubating for 10 min at 65 °C. cDNA was synthesized following the supplier’s protocol, adding 1.5 μL of 300 IU MMLV (28025-013, Invitrogen, Carlsbad, CA, USA), 6 μL 5X First-Strand Buffer, 1.5 μL 10 mM dNTPs(10297-018, Invitrogen, Carlsbad, CA, USA), 0.1 μL Random Primers(48190011; Invitrogen, Freder-ick, MD, USA, 3 μL 0.1 M DTT, 1 μL RNaseOUT™ Recombinant Ribonuclease Inhibitor (40 units/μL) (10777-019, Carlsbad, CA, USA), and H2O for a total 30 μL reaction. For human samples, 50, 25, and 12.5 ng of Poly A+ mRNA was similarly treated.

Expression was detected by qPCR using 1 μL of the cDNA reaction plus 6 μL 2x TaqMan Gene Expression MasterMix (4369016 Applied Biosystems, Foster City, CA, USA) and 6 μL diluted primers in 96 well-plates in a 7500 Real-Time PCR System (4351105, Applied Biosystems, Foster City, CA, USA). As control for general gene ex-pression, we used human negative controls of the reverse-transcription step (all reagents and RNA samples but without reverse transcriptase) and the PCR step (all reagents but no reverse-transcribed samples) were included in each assay plate. We used the house-keeping gene (TBP) as an expression control gene since we have previous experience in human tissue samples [34,35]. The following genes were determined: *VEGFA*: Vascular endothelial growth factor A; *IGF1*: Insulin-like growth factor I; *NOS3* or *eNOS*: Nitric oxide synthase 3 or endothelial NOS; *MSTN*: Myostatin; *NOX4*: NADPH (Nicotinamide adenine dinucleotide phosphate oxidase) 4; *MYOG*: Myogenin; *KDR*: VEGFA receptor 1; *IL6*: Interleukin 6; and *TNF*: Tumor Necrosis Factor. All of them appear in italic in the text and Figures.

ΔCt relative values with respect to the commercial pool of human muscle were calculated for each gene related to TBP. When compared from time zero (control time) to two months of treatment/placebo, ΔΔCt values were obtained. Primer sets and TaqMan assays (Applied Biosystems) used are shown in Table 1.

### 2.5. Statistical Analysis

Mean, median, and standard deviation and standard error values were calculated for each group. Serum biomarkers and molecular data were analyzed at baseline, and for differences between basal and final time points in the two treated groups. Results were also stratified by patients with or without DM, but not by type of DM.

Data were processed with GraphPad prism^®^ v8 software (San Diego, CA, USA). The initial step was to check if data within a group followed a normal distribution and if their variances were equal or not. For normality distribution, we used the Shapiro–Wilk test (as the sample size was less than 50) and Fisher’s test for variances. Based on the results, if the groups were normal and homoscedastic, we used t-test; if the groups were normal and not homoscedastic, we used *t*-test with Welch’s correction; finally, if the groups followed a non-normal distribution, we used the test of Wilcoxon–Mann–Whitney. To compare among qualitative, or quantitative to qualitative variables, X^2^ test was performed with Fisher’s test when expected frequency was less than 5.

Correlations (r) were measured using the SPSS^®^ software v27 (Armonk, NY, USA), mainly the Spearman correlation coefficient for non-normal qualitative or quantitative variables. The graphs were constructed by using the mentioned Graphpad prism^®^ software. In addition, Kaplan–Meier and AUC–ROC curves (AUC: Area Under the Curve; ROC: Receiver Operating Characteristics) were carried out by using R statistics software (version 4.0.3, free software). Fisher and Mantel–Haenszel tests were used for odds ratios analysis.

In addition to the univariate study, a multivariate study was addressed by using multiple logistic regression analysis.

## 3. Results

### 3.1. General Characteristics

The baseline characteristics between both groups of the study were similar in terms of age, sex, hypertension (HT), DM with or without neuropathy, chronic kidney disease (CKD), dialysis, heart disease, or Rutherford class. However, on the one hand, some differences were detected between groups on tobacco consumption, with a higher number of nonsmokers in the intervention group (GH: 88.89% vs. placebo: 50%, *p* = 0.0107). On the other hand, the presence of trophic lesions in the foot was more frequent in the GH group than in the control group (GH: 83.3% vs. placebo: 50%, *p* = 0.0381). In CLTI studies, a higher proportion of men than women is usually found, as in our study (men/women: 79.4% vs. 20.6%), since this disease has a higher incidence in men, without any difference between GH-treated patients and controls (*p* = 0.2715). Our group previously published a table with all these characteristics (see Table 2 in [28]). In the sample analyzed for this study, 59% of patients suffered from DM, 57.6% of them with established neuropathy. All diabetic patients were type II, and all except one suffered from DM of more than 10 years of evolution. DM affected 53.3% of the men and 100% of the women in this study. Age was stratified into three groups (<65 years; 65–80 years; and >80 years). The percentage of diabetic patients in each age group was as follows: <65: 29.2%; 65–80: 33.3%; >80: 37.5% (*p* = 0.2531).

### 3.2. Hemodynamic Parameters and Plasma Biomarkers

The baseline characteristics (mean and median) of the hemodynamic and plasma parameters are schematized in Table 2: Age: 71.5 ± 12.4 years; ankle–brachial index (ABI): 0.23 ± 0.23; ankle pressure (AP): 38.6 ± 37.02; TNF-α: 10.66 ± 4.9 pg/mL; hsCRP: 1.6 ± 2.2 mg/dL; CyC: 1.45 ± 0.9 mg/L; B2M: 0.4 ± 0.3 mg/L; fibrinogen: 560.21 ± 128.51 mg/dL; HbA1C: 6.5 ± 1.02%; neutrophil-to-lymphocyte ratio (NLR): 3.5 ± 2.8; IGF-1: 134.9 ± 53.2 ng/ml; and IGF-1BP3: 3.06 ± 1.1 μg/mL.

An elevated level of plasma TNF-α (≥8.1 pg/mL) was seen in 68.57% of patients (GH: 83.3%; Placebo: 52.9%), while a high level of hsCRP (≥0.5 mg/dL) was detected in 60.5% (GH: 70%; Placebo: 50%). An elevated NLR (≥2.5) appeared in 52.6% of patients, and CyC was also increased in the GH group compared with the placebo. The plasma levels of IGF-1 increased in 8.11% of patients, mainly in the GH group, and the plasma levels of IGFBP3 were found low in 44.1% of patients: 45.5% vs. 42.9%, GH and placebo, respectively (*p* = 0.634).

Table 3 depicts relevant information about the groups. As can be seen, the level of plasma biomarkers of inflammation such as TNF-α, hsCRP, and CyC was significantly higher in the GH group at baseline, which implies a higher inflammatory state in this group. Even basal levels of fibrinogen were higher in the GH group (601.25 vs. 489.85, *p* = 0.0425), which supports this statement. At the end of the study, only plasma TNF-α was significantly decreased compared to the placebo, as previously reported by our group [28].

### 3.3. Basal mRNA Expression

**Figure 1:**
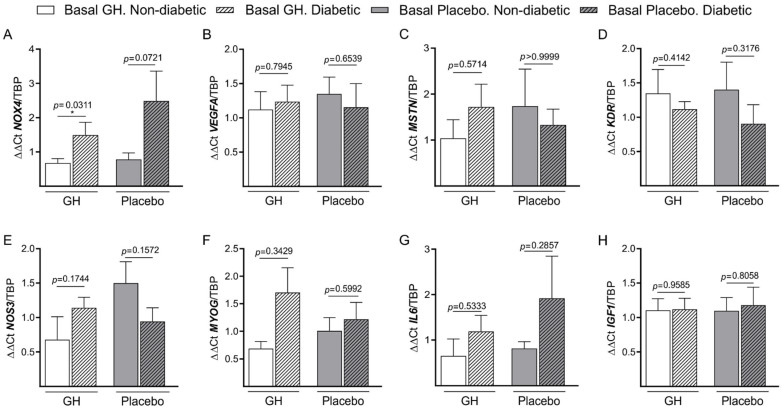
**mRNA expression of different genes at baseline in both groups, GH, and placebo) in DM and non-DM patients**. (**A**). *NOX4*: NADPH (Nicotinamide adenine dinucleotide phosphate oxidase) 4; (**B**). *VEGFA*: Vascular endothelial growth factor A; (**C**). *MSTN*: Myostatin; (**D**). *KDR*: VEGFA receptor 1. (**E**). *NOS3* or *eNOS*: Nitric oxide synthase 3 or endothelial NOS; (**F**). *MYOG*: Myogenin; (**G**). *IL6*: Interleukin 6; (**H**). *IGF1*: Insulin-like growth factor I; GH: Growth hormone; Placebo: control group; Basal: Baseline. * *p* < 0.05.

After analyzing the basal mRNA expression of different genes in the skeletal ischemic muscle (Figure 1A–H), it was found that only *NOX4* had a consistently higher expression in the group with DM and GH compared to the group without DM and placebo (*p* = 0.0346) (Figure 1A).

### 3.4. Basal and Final mRNA Expression

**Figure 2:**
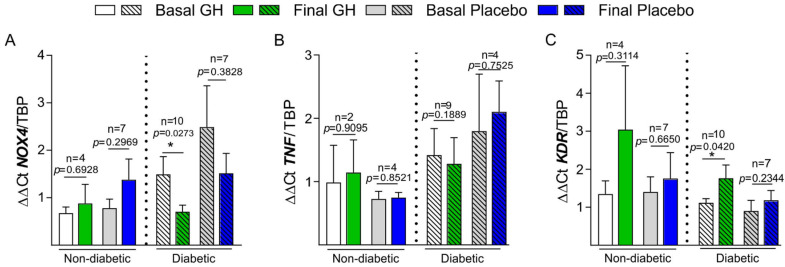
**Basal to final mRNA expression of different genes.** *NOX4* (**A**), *TNF* (*TNF*-α) (**B**), and *KDR* (**C**). Findings in both groups, GH, and placebo, with DM and without DM. * *p* < 0.05.

*NOX4* mRNA expression underwent a real decrease in DM ischemic patients treated with GH compared to non-DM (*p* = 0.0348) (Figure 2A). *TNF* mRNA expression (*TNF-α*) was not reduced in the GH group, neither in patients with DM nor in those without DM (Figure 2B). The angiogenesis marker *KDR* underwent a real increase in its expression during GH treatment, which was only significant in patients with DM (*p* = 0.036) (Figure 2C).

### 3.5. Plasma Biomarkers and mRNA Expression

**Figure 3:**
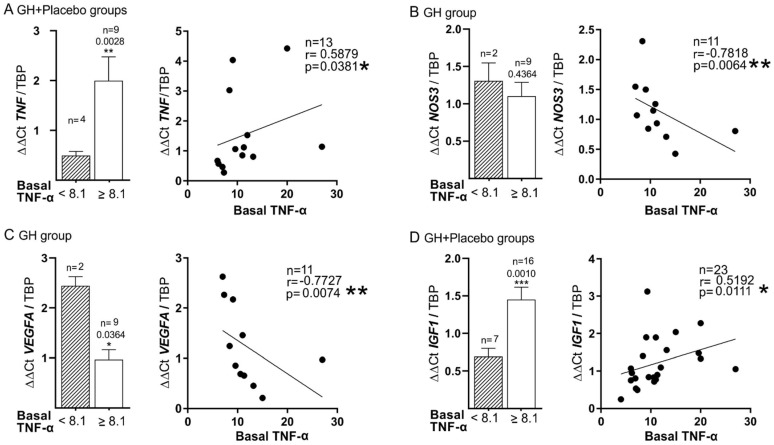
**Baseline plasma levels of TNF-α related to different muscle gene expressions in DM patients.** Both groups: GH and placebo. Correlation between plasma TNF-α and muscle *TNF* (**A**), *NOS3* (**B**), *VEGFA* (**C**), and *IGF-1* (**D**) mRNA expressions. TBP: housekeeping gene used as an expression control gene. * *p* < 0.05; and ** *p* < 0.001.

Figure 3 depicts the relationship between basal plasma TNF-α and gene expression in ischemic skeletal muscle for DM patients in both groups of treatment (GH and placebo). First, a positive correlation was found between plasma TNF-α level and mRNA expression of *TNF-α* (r = 0.588, *p* = 0.035) (Figure 3A); this was especially relevant for those patients with TNF-α ≥8.1 (r = 0.802 *p* = 0.001) (see also Table 4 and Table 5). Second, there was an inverse correlation between the expression of *TNF-α* and *NOS3* (r = −0.4999, *p* = 0.0151), more evident in the GH group (r = −0.78, *p* = 0.0064) (Figure 3B). Third, an inverse correlation was found between plasma TNF-α and the angiogenic factor *VEGFA* (r = −0.4321, *p* = 0.0395), also stronger in the GH group (r = −0.7727, *p* = 0.0074) (Figure 3C). Fourth, the high level of inflammation, expressed by plasma TNF-α ≥8.1, was related to the expression of *IGF-1* (*p* = 0.0010), showing a moderate but positive correlation between both (r = 0.5192, *p* = 0.0111) (Figure 3D). All these relationships, as mentioned above, appeared exclusively or were stronger in patients with DM, independent of the treatment received.

**Figure 4:**
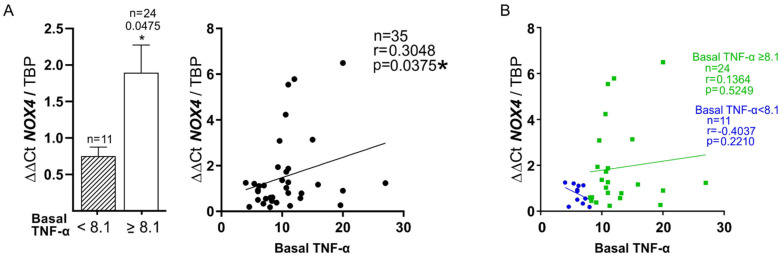
**Relationship between high levels of plasma TNF-α (≥8.1 pg/mL) and *NOX4* expression in all patients of the study**. (**A**). Left: expression of muscle *NOX4* mRNA in relation to plasma TNF-α. Striped bar: TNF-α < 8.1 pg/mL. White bar: TNF-α ≥ 8.1 pg/mL. Right: Spearman correlation between both markers. (**B**). Trend of this correlation differentiating by TNF-α values: ≥8.1 (green color) and <8.1 (blue color). * *p* < 0.05.

In addition, those patients with the highest levels of plasma TNF-α at baseline had higher levels of redox stress (*p* = 0.0475), with a weak but significant correlation between plasma TNF-α and *NOX4* (r = 0.35, *p* = 0.0375) (Figure 4A). Interestingly, the latter relationship shows different behavior depending on the levels of plasma TNF-α (≥8.1 or <8.1) (Figure 4B). It should be noted that patients without DM showed the best correlation between both markers (0.645, *p* = 0.032), especially when plasma TNF-α reached ≥ 8.1 pg/mL (r = 0.717, *p* = 0.009) (see Table 5).

**Figure 5:**
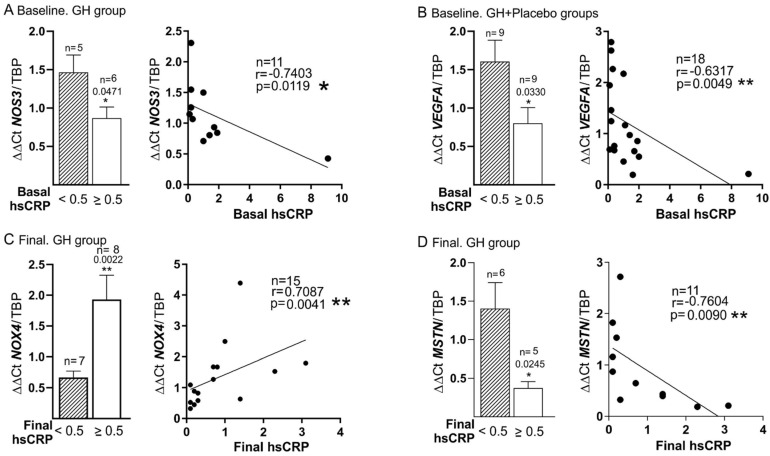
**Correlation between plasma hsCRP and muscle gene expression in diabetic patients.** (**A**). Baseline expression levels of hsCRP and *NOS3* in the GH group. (**B**). Baseline hsCRP and *VEGFA* levels in both groups. (**C**). Final hsCRP and redox stress levels measured by final *NOX4* in the GH-treated patients. (**D**). Final hsCRP levels were related to final *MSTN* mRNA expression in GH group. Striped bars: hsCRP < 0.5; White bars: hsCRP ≥ 0.5. * *p* < 0.05; and ** *p* < 0.001.

High levels of hsCRP were related to *NOS3* expression at baseline (r = −0.74, *p* = 0.009), also in DM patients treated with GH (Figure 5A), while at the end of the study (final time point) a value of hsCRP ≥ 0.5 was negatively correlated to muscle *NOS3* expression, both in patients with DM and without DM treated with GH (r = −0.711, *p* = 0.021) (see Table 4)

Basal levels of hsCRP were negatively associated with basal *VEGFA* expression too in both groups of treatment, although more specifically in DM (r = −0.632, *p* = 0.005) and for those patients with the highest levels of inflammation measured by hsCRP ≥ 0.5 (*p* = 0.0330) (Figure 5B). In the GH group, the final hsCRP was also related to the final expression of *NOX4* (r = 0.7, *p* = 0.0041) (Figure 5C), while its relationship with *MSTN* expression was negative (r = −0.7604, *p* = 0.009) (Figure 5D).

**Figure 6:**
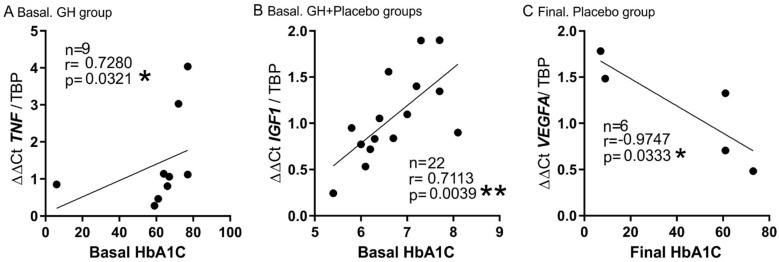
**Correlation of plasma HbA1C and muscle mRNA expression in diabetic patients.** (**A**). *TNF*. (**B**)**.** *IGF-1*. **C.** *VEGFA*. (**A**,**B**) represent correlations at baseline, while (**C**) represents final time point. * *p* < 0.05; ** *p* < 0.001.

Of interest are also the findings of plasma HbA1C correlations at baseline. Indeed, HbA1C was highly correlated to *TNF-α* mRNA expression in the GH group with DM (r = 0.7280, *p* = 0.0321) (Figure 6A). The same occurred between HbA1C and *IGF-1* mRNA expression in patients with DM, but, in this case, in both groups of patients (GH and placebo, r = 0.7, *p* = 0.0039) (Figure 6B). At the end of the treatment, the final levels of HbA1C were also inversely related to the final muscle expression of *VEGFA* in DM, but only in the placebo group (r = −0.9747, *p* = 0.0333) (Figure 6C).

As our group previously published, *NOX4* expression was specifically reduced in the GH-treated group (*p* = 0.025), while it tended to increase in the placebo group (see Figure 5 in [28]).

All correlations between plasma markers and mRNA expression of different genes are summarized in Table 4 (*NOS3*, *VEGFA*, and *TNF-α*) and **5** (*IGF-1*, *MSTN*, *MYOG*, *KDR*, and *NOX4*).

### 3.6. Mortality in the GHAS Trial

Special attention should be paid to mortality in the GHAS trial. The cumulative mortality at 12 months reached 47.4% of CLTI patients, being higher in GH-treated patients than in patients receiving placebo, both at 2 months (5.5% vs. 0%, *p* = 0.42) and at 12 months (29.4% vs. 12.5%, *p* = 0.23) (Table 6).

**Figure 7:**
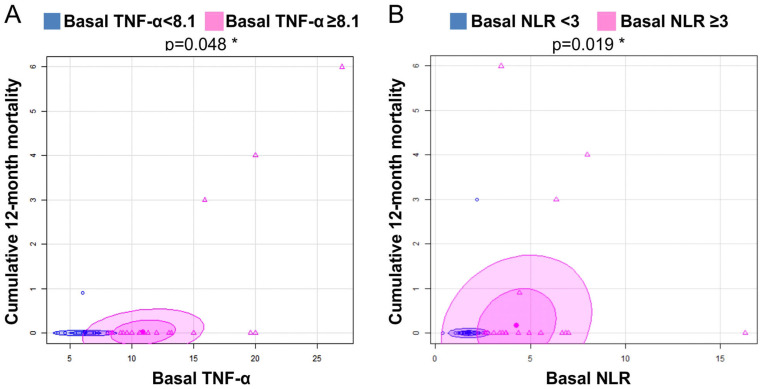
**Cumulative 12-month mortality and plasma markers**. (**A**). Basal TNF-α; (**B**). Basal NLR (Neutrophil-to-lymphocyte ratio). * *p* < 0.05.

In order to identify some possible predictors associated with mortality in the GHAS trial, we performed a careful analysis of variables that could be responsible for it. We found that a value of plasma TNF-α ≥ 8.1 pg/mL was significantly associated with cumulative mortality at 12 months or long-term mortality (*p* = 0.0487, OR = 2.5, CI (95%): 0.23–136.6, Fisher test: *p* = 0.63) (Figure 7A). In addition, a value of NLR ≥ 3 was also associated with long-term mortality (*p* = 0.019, OR = 6.9, CI (95%): 0.71–353.7, X^2^ with McNemar correction: *p* = 0.01946, Fisher test: *p* = 0.093) (Figure 7B). For NLR ≥ 3, the Mantel–Haenszel test showed no influence of other variables such as smoking, DM, hsCRP, and TNF-α on the results.

**Figure 8:**
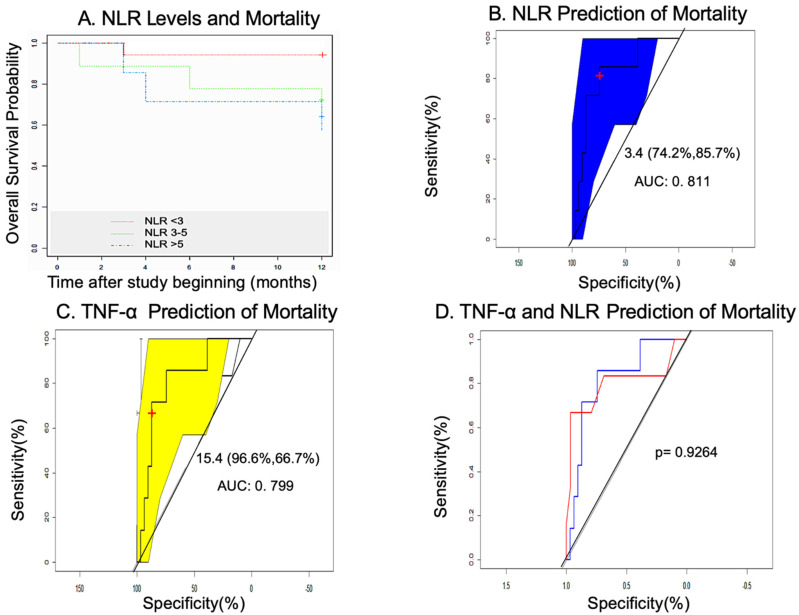
**Relationship between NLR and plasma TNF-**α **with mortality.** (**A**). Kaplan-Meier analysis of NLR and mortality. (**B**). AUC-ROC (Area Under the Curve-Receiver Operating Characteristics) curve of NLR related to mortality. (**C**). AUC-ROC curve of plasma TNF-α related to mortality. Color blue and yellow represent confidence intervals. Values in brackets represents sensitivity and specificity, respectively (**D**). Comparation between plasma TNF-α and NLR as predictors of mortality showed no significant differences (*p* = 0.9264).

The Kaplan–Meier survival analysis indicated that patients with NLR ≥ 3 and NLR ≥ 5 had a higher mortality rate (Figure 8A). AUC–ROC curves identified the best cutoff point for NLR to predict mortality in 3.4 (74.2%, 85.7%), with an AUC value of 0.811 (0.733–0.857) and a power of 0.77 (Figure 8B). The best cutoff point for plasma TNF-α was 15.4 (96.6%, 66.7%), with an AUC value of 0.7989 (52.4%–100%) and a power of 0.67 (Figure 8C). After comparing both biomarkers in terms of their predictive ability, we observed that there were no statistical differences between them (*p* = 0.9264) (Figure 8D).

Some clinical predictors of long-term mortality were also found, such as chronic obstructive pulmonary disease (COPD) (*p* = 0.042, OR = 5.8, CI (95%): 0.84–40.7) and an American Society of Anesthesiologists class 4 (ASA 4) compared to a class 3 (ASA 3) (*p* = 0.0119, OR = 15.75, CI (95%): 0.87–284.9). (Table 7).

**Figure 9:**
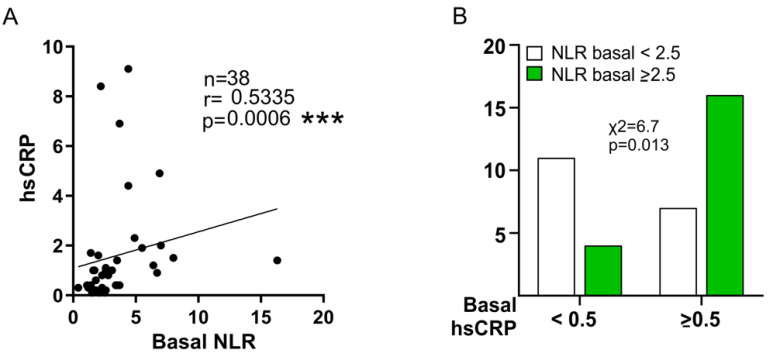
**Relationship between basal levels of NLR and plasma hsCRP.** (**A**). Spearman correlation. (**B**). Histogram comparing hsCRP ≥ 0.5 and NLR ≥ 2.5. Green bars: NLR ≥ 2.5; White bars: NLR < 2.5. *** *p* < 0.0001.

It is also noteworthy that the basal level of hsCRP was related to short-term mortality (2 months) (*p* = 0.008). In addition, the level of NLR at baseline was closely related to the level of hsCRP (r = 0.5335, *p* = 0.0006) (Figure 9A), especially for the values of hsCRP ≥ 0.5 and NLR > 2.5 (*p* = 0.013) (Figure 9B).

### 3.7. Statistical Study: Measures of Association of Variables

#### 3.7.1. Univariate Analysis

**Figure 10:**
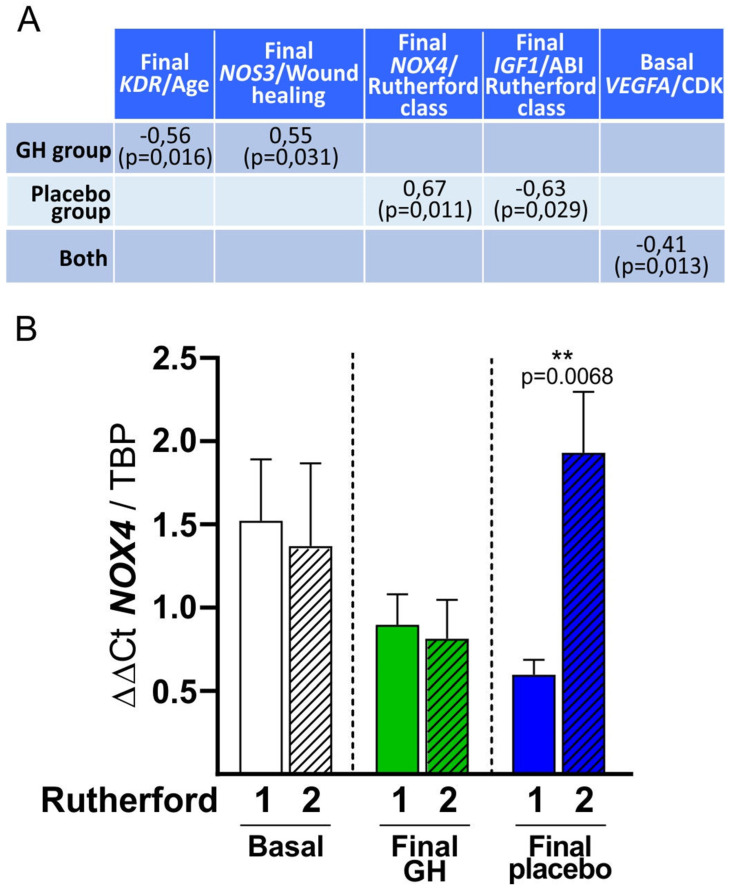
(**A**): Relation between different muscle genes and clinical variables in both groups. (**B**): Graph showing the link between *NOX4* mRNA expression and Rutherford class, grouped in 1: less severity of ischemia, and 2: more severity of ischemia. White box: basal level of *NOX4*; Green bars: final level of *NOX4* in the GH group; and Blue bars: final level of *NOX4* in the placebo group. Striped bars: group with severe ischemia. ** *p* < 0.001.

The linear regression model showed that values of hsCRP ≥ 0.5 mg/dL at baseline tended to be related to the cumulative amputation ratio at 12 months (*p* = 0.056). The analysis of the COR curves indicated that the best cutoff point was 0.7mg/dL (AUC: 66.4%, 49.3–83.5%). However, the statistical power of this association was low (0.2579), therefore a larger sample size is needed to confirm this possible relation. In the placebo group, final levels of *NOX4* were significantly related to the Rutherford class of patients (r = 0.67, *p* = 0.011), while final *IGF-1* was related to ABI (r = −0.63, *p* = 0.011). In the GH group, the final level of *KDR* was affected by age (r = −0.56, *p* = 0.016), while the expression of *NOS3* correlated positively to wound healing (r = 0.55, *p* = 0.031). At baseline, *VEGF-A* expression was related to CKD (r = −0.41, *p* = 0.013) (Figure 10, top). The specific relationship between *NOX4* expression and Rutherford class is represented in Figure 10, bottom.

#### 3.7.2. Multivariate Analysis

The multiple logistic regression model also demonstrated the relationship between basal NLR ≥ 2.5 and hsCRP ≥ 0.5 (*p* = 0.013), which was confirmed by using Kendall’s Tau (0.41, *p* = 0.010).

However, high levels of plasma TNF-α (≥8.1) at baseline were not related to anything using the multivariate analysis, although there was a trend to be related to basal hsCRP ≥ 0.5 (*p* = 0.055), NLR (*p* = 0.063), and CKD (*p* = 0.059). Basal hsCRP ≥ 0.5 was also not related to anything with this model, although there was a tendency to be associated with the Rutherford class (*p* = 0.055).

## 4. Discussion

In this study we analyzed the role of some plasma biomarkers and the expression of some muscle genes in the ischemic muscle of PAD patients, focusing on the data from the GHAS trial carried out in the special group of patients suffering from CLTI, which shows a high level of inflammation and, in parallel, a high rate of morbidity and mortality [32]. Most studies on this field have investigated plasma samples from patients with intermittent claudication, a less severe form of PAD, without information on the status of the ischemic muscle. Therefore, the real role of inflammatory cytokines in advanced PAD needs to be better defined. In the GHAS trial, we cross-linked plasma information with that obtained from gene expression at the level of ischemic skeletal muscle of the lower extremities.

Our data confirm the high level of inflammation in CLTI patients (Table 2) and the high mortality rate of this special population (Table 6). Interestingly, according to plasma biomarker data, patients in the GH group were significantly more affected by inflammation than those in the placebo group (Table 3). The high level of inflammation in GH-treated patients configured a severely ill population with higher levels of redox imbalance, biomarkers, genetic alterations, and mortality, as shown in the results, especially if they suffered from DM. This is also consistent with the fact that patients in the GH group had significant higher levels of fibrinogen and, as published by our group [28], more trophic lesions in the foot than those observed in the placebo group, indicating a more severe stage of ischemia. Nevertheless, at the end of the study, only plasma TNF-α experienced a significant reduction in GH-treated patients [28], which is consistent with previous studies [36,37]. This supports both the anti-inflammatory action of GH and the key role of TNF-α in PAD. The effect of GH on plasma hsCRP did not reach a significant reduction, probably as a consequence of the relatively small sample size studied. However, the MESA trial or Mendelian randomization studies have shown neutral results of plasma hsCRP in PAD [38], considering CRP an acute phase protein rather than a causal factor [20]. As we can see from our results, this statement might not be completely true for CLTI patients, as we found how hsCRP could influence on the gene expression of both processes: angiogenesis (*NOS3*, *VEGFA*) and redox balance (*NOX4*) (Figure 5A–C).

Again, we demonstrated here that oxidative stress plays a crucial role in CLTI, as *NOX4* was the only gene that our group found significantly increased in patients with high levels of inflammation and DM (Figure 1A), which supports the link between inflammation and DM, as it was advanced in a previous publication [28].

Despite the fact that *NOX4* was significantly reduced in all GH-treated patients, our data show that patients with DM benefited more compared to non-DM patients (*p* = 0.0348) (Figure 2A).

Although GH decreased plasma TNF-α, a parallel significant reduction in mRNA expression of this marker (*TNF*) was not detected in the ischemic muscle, neither in patients with DM nor in patients without DM (Figure 2B). The possible explanation could be related to the possibility that GH reduces the cleavage of that TNF-α joined to the cell membrane, which is the source of soluble TNF-α, hence lowering plasma TNF-α, but it would not decrease the expression of *TNF* at the cell membrane [39]. The binding of TNF-α to the cell membrane depends on cell–cell interactions, and, furthermore, there are many polymorphisms of the *TNF* gene, both functional and structural, that affect gene function, mRNA production, and its final expression [39]. The complex regulation of the *TNF* gene and the lack of information about the behavior of this gene in clinical studies with ischemia, among others, might explain this finding.

What is also of great relevance is the negative correlation between plasma levels of TNF-α and hsCRP with mRNA expression of *NOS3* and *VEGFA*, indicative of the deleterious effect of these cytokines on tissue regeneration related to angiogenesis (Figure 3 and Figure 5). On the one hand, the role of plasma TNF-α in this setting was expected, as it was advanced in a previous experimental study [40]. Now our group first confirms that this important fact also occurs in humans with CLTI. Nevertheless, the possible role of CRP on angiogenesis was surprising. The data obtained in this regard confirm that this protein could be associated with homeostasis and angiogenesis in the state of high levels of inflammation, as indicated by an experimental study [41]. Therefore, it could be considered that this protein might have an active role rather than that of a passive acute phase marker. Both biomarkers, TNF-α and hsCRP, were also negatively correlated to *VEGFA* expression, supporting their impact on angiogenesis. Other authors have found a relationship between plasma levels of VEGF and hsCRP in experimental models of stroke [42]. Again, our group is the first to describe the connection between the expression of *VEFGA* in the skeletal muscle and plasma hsCRP in patients with lower limb ischemia.

The mentioned findings are parallel and consistent with the positive significant correlation between basal plasma TNF-α and *NOX4* in all patients of this study. The data obtained show that plasma TNF-α has a different behavior pattern depending on its levels related to *NOX4* expression (Figure 4B), which supports the fact that a high level of TNF-α, and hence, of inflammation, is responsible for its deleterious effect on redox balance. The fact that patients without DM seemed to have a greater correlation between TNF-α and *NOX4* needs confirmation and explanation.

In line with this finding, the use of TNF-α inhibitors in patients with a high level of inflammation, such as rheumatoid arthritis, decreased atherosclerotic CV events similarly to the use of IL-6 blockers [43], also improving the endothelial dysfunction [24]. These drugs have demonstrated an anti-inflammatory efficacy by decreasing the expression of several molecules [25,44]. However, the benefit of TNF-α inhibitors for PAD management seems to be determined by *TNF* gene polymorphisms, and pharmacogenetics could help identify which individuals benefit most from them [45].

One of the most important data obtained in our study is the link between the plasma levels of TNF-α and *NOS3* expression. As previously published, *NOS3* is one of the most important enzymes that maintains vascular homeostasis, and it is involved in vascular defense against chronic or excessive inflammation [27,28]. TNF-α can affect the activity of *NOS3* with an independent action on its gene promoter in a dose- and time-dependent manner [40]. This proinflammatory molecule facilitates the phosphorylation of *NOS3*, reducing the level of nitric oxide (NO), facilitating endothelial dysfunction, and altering the regeneration process [40,46]. This finding in humans suggests that TNF-α might be an accurate target for CLTI. To see the importance of the decrease in plasma TNF-α by GH, it is worth highlighting a meta-analysis of 54 prospective cohort studies on inflammation and PAD, in which plasma TNF-α was able to predict the risk of CV events with the same magnitude as the therapies for lowering blood pressure or lipids [47,48].

The observation that plasma TNF-α and *NOX4* expression run in parallel is also remarkable. NOX4 enzyme is the main isoform of NADPH oxidase responsible for TNF-α-induced oxidative distress and apoptosis of different cells in the body [49,50,51]. An important source of ROS in blood vessels comes from NOX catalytic enzymes, which are ubiquitously distributed in the three vessel layers. NOX4 enzyme, which maintains a physiological basal ROS generation, is highly expressed in cells under stress [27]. This protein could play a fundamental role in the regulation of angiogenic growth factors, such as VEGFA, since the inhibition of NOX and/or the production of mitochondrial ROS decreases the expression of this growth factor [52]. Thus, our finding of the link between TNF-α and *VEGFA* at baseline in those patients with a high level of inflammation (GH group) makes sense. Although TNF-α can favor vascular homeostasis when it is produced in small concentrations by endothelial cells (ECs), a chronically high production of this particle determines deleterious effects, overstimulating NOX4 enzyme, which eventually leads to an excess of ROS that ends up in the final inactivation of NO [22,53]. These circumstances warranted ECs activation, favoring the prothrombotic state and thrombotic complications associated with advanced PAD [29,54].

While in the CANTOS study the relevant role is for IL-6 and IL-1B in patients with ischemic heart disease [20], Gardner et al. demonstrated that in patients with PAD, TNF-α and IL-8 levels were increased but not IL-6 levels [22], which supports the fact that both diseases seem to show differences in the inflammatory pattern. However, Gardner’s study was conducted in patients with intermittent claudication, a less severe form of ischemia with less inflammation, and the causal association between this or other biomarkers and PAD has not yet been established. In the GHAS trial, *IL-6* mRNA expression was not altered at baseline, and GH treatment did not affect this expression either. However, information on plasma IL-6 levels was not included in the GHAS study.

Baseline level of plasma HbA1C appears to be well correlated with TNF-α in the GH group with DM, supporting the role of this biomarker in patients with a high level of inflammation (Figure 6A). The final levels of HbA1C were inversely related to *VEGFA* mRNA expression in DM, but only in the placebo group (r = −0.9747, *p* = 0.0333) (Figure 6C), which means that untreated DM patients maintain a higher level of inflammation and, therefore, angiogenesis is negatively affected. Thus, HbA1C should be a possible target in these patients, highlighting the need to a good glycemic control.

On the other hand, mRNA expression of *IGF-1* seemed to follow an inverse behavior, increasing its expression in ischemic muscles with inflammation, probably as a defense mechanism. That is, in cultures of ECs with a high level of inflammation induced by the addition of CRP, this protein increases the phosphorylation of the enzyme eNOS, decreasing its activity. When IGF-1 protein is added to this medium rich in CRP, the activity of the enzyme eNOS increases [41], which means that IGF-1 protein exerts a negative feedback on the pernicious effect of CRP on ECs, and that these cells can produce IGF-1 in response to a high level of CRP. In our trial, the treatment with GH restored inflammatory and redox imbalances, and the association between CRP and IGF-1 reverted to inverse (Table 5). In the placebo group, this benefit did not appear, maintaining the positive association. Although CRP and IGF-1 were investigated in PAD–CLTI patients in a prior study [55], only the plasma level of these factors was studied and not the gene expression in the ischemic muscle.

Last but not least, we obtained relevant data on some plasma biomarkers and mortality. NLR has been proposed as a marker of adverse complications in CV disease, malignancy, and infection [56]. In PAD, NLR was incorporated into the ERICVA score for the prediction of poor prognosis in patients with CLTI [57]. In our study, we saw that NLR ≥ 3 at baseline was related to cumulative mortality at 12 months (Figure 8A) and was correlated with the level of plasma hsCRP (Figure 9). Although the appropriate cutoff point for NLR in CV disease has yet to be adequately defined [26], we found that a value of 3.4 was the best predictor for long-term mortality in CLTI patients (Figure 8B) with a good sensitivity and specificity and a light to moderate power of the test. This observed value was very close to the average NLR value in the series (3.5). Perhaps, in studies with acute limb ischemia, where NLR is normally higher, the threshold seems to be 5.4 [56], and in those studies performed in patients with intermittent claudication, the cutoff point was 5 [57]. It makes sense that the higher the level of inflammation, the lower the NLR cutoff point could be for predicting mortality, and that this cutoff point might be different depending on the morbid condition and the level of inflammation. Plasma TNF-α ≥ 8.1 was also found as a possible predictor of mortality. The calculation of the confidence interval for the odds ratio of both biomarkers did not find significant differences (see CI in Table 7), probably as a consequence of the small sample size. However, in the case of NLR, it can be seen a clear trend.

The main limitation of the GHAS trial is the relatively small sample size and the lack of confirmation of some of these data (with some exceptions) when the multivariate model was used for statistics, although the latter was only applied to cross-link clinical and plasma variables, not for gene expression. Furthermore, as this study represents the first clinical trial exploring the use of GH for angiogenesis, the dose and time of application of this hormone have not properly been established [28,29]. However, plasma and gene expression data at baseline are not affected by the intervention in this study. The main advantage of this study is that the ischemic muscle has been studied in depth, offering relevant data.

## 5. Conclusions

Vascular homeostasis and redox balance govern vascular health or disease states. Chronic limb-threatening ischemia is responsible for a high level of inflammation and mortality rate and seems to show differences in the inflammatory pattern compared to ischemic heart disease. The behavior of some biomarkers in patients with lower limb ischemia depends on the level of inflammation. Plasma TNF-α plays a crucial role in both inflammation-mediated redox distress and endothelial dysfunction and regeneration, affecting *NOS3* and *VEFGA* expression. CRP seems to play a more active role than previously assigned, affecting also redox balance and angiogenesis. Muscle NOX4 enzyme has been revealed as key for redox imbalance, and IGF-1 could be produced as a mechanism of defense against ischemia. Diabetic patients with ischemia of the lower limbs represent an especially targeted population for antioxidant and regenerative therapies as GH. NLR ≥ 3, together with plasma TNF-α ≥ 8.1, could be a good predictor of mortality in this morbid condition.

All these findings are clinically relevant and open new paths in the search for new drugs to decrease cardiovascular events and to relieve symptoms in patients with chronic limb-threatening ischemia. Our results are novel because they represent the link between plasma and muscle gene expression in humans with limb ischemia, but need to be corroborated in a larger clinical trial.

## Figures and Tables

**Table 1:** **Real-time PCR (RT-qPCR).** Primer sets, TaqMan assays and conditions used for RT-qPCR in the GHAS trial for muscle gene expression analysis. Genes are written in italic.

Gene	Sequence	Amplification Size	Annealing Tª
*TBP*	Fw: 5′-GCCCGAAACGCCGAATAT-3′	67 bp	60 °C
	Rv: 5′-TTCGTGGCTCTCTTATCCTCATG-3′		
	Pb: 5′-TCCCAAGCGGTTTGCTGCGGTA-3′		
*VEGFA*	Applied Biosystems: Hs00900055_m1	67 bp	60 °C
*IGF1*	Applied Biosystems: Hs01547656_m1	68 bp	60 °C
*NOS3*	Applied Biosystems: Hs01574665_m1	86 bp	60 °C
*MSTN*	Applied Biosystems: Hs00976237_m1	69 bp	60 °C
*NOX4*	Applied Biosystems: Hs01379108_m1	64 bp	60 °C
*MYOG*	Applied Biosystems: Hs01072232_m1	76 bp	60 °C
*KDR*	Applied Biosystems: Hs00911700_m1	83 bp	60 °C
*IL6*	Applied Biosystems: Hs00174131_m1	95 bp	60 °C
*TNF*	Applied Biosystems: Hs00174128_m1	80 bp	60 °C

**Table 2:** **Mean and median results of inflammatory parameters in CLTI patients from the GHAS trial.** TG: Triglycerides; HDLc: High density lipoprotein cholesterol; LDLc: Low density lipoprotein cholesterol; ABI: Ankle–brachial index; AP: Ankle pressure; IGF-1: Insulin-like growth factor 1; IGFBP3: IGF-1 binding protein 3; TNF-α: Tumor necrosis factor alpha; hsCRP: C-reactive protein of high sensitivity; B2M: Beta-2 microglobulin; CyC: Cystatin C; HbA1C: Glycosylated hemoglobin; NLR: Neutrophil-to-lymphocyte ratio; Fibrin.: Fibrinogen; SD: Standard deviation; Min: Minimum; and Max: Maximum.

	TG	HDLc	LDLc	ABI	AP	Age	IGF-I	IGFBP3	TNF-α	hsCRP	B2-M	CyC	HbA1C	NLR	Fibrin.
**Mean**	164.1	45.5	97.9	0.23	38.6	71.5	134.9	3.06	10.66	1.6	0.4	1.45	6.5	3.5	560.21
**Median**	132.5	41	100	0.2	31.5	72	125	2.9	10	0.95	0.3	1.2	6.3	2.6	505
**SD**	88.1	15.1	32.1	0.23	37.02	12.4	53.2	1.1	4.9	2.2	0.3	0.9	1.02	2.8	128.51
**Mín.**	46	29	36	0	0	49	38	0.5	4	0.1	0.14	0.55	5.1	0.4	403
**Max.**	412	97	161	0.93	140	93	275	5.2	27	9.1	1.1	3.8	8.9	16.3	853

**Table 3:** **Biomarker distribution in the GHAS study**. Basal: Baseline or pretreatment values; Final: Final values after 2 months of treatment. Statistical significance is highlighted in colors. GH: Growth hormone; Obs: Observed; SD: Standard deviation; TNF-α: Tumor necrosis factor; hsCRP: C-reactive protein of high sensitivity; B2M: Beta-2 microglobulin; and CyC: Cystatin C. * *p* < 0.05.

Plasma Marker		GH			Placebo		
	Obs.	Mean	SD	Obs.	Mean	SD	*p*-Value
**TNF-α (Basal)**	16	12.35	5.2	16	8.78	3.9	0.0184 *
**TNF-α (Final)**	15	10.93	5.12	14	8.04	3.6	0.0464 *
**hsCRP (Basal)**	18	2.07	2.86	16	0.79	0.70	0.0454 *
**hsCRP (Final)**	17	1.1	1.38	14	3.42	7.51	0.2188
**B2M (Basal)**	7	0.47	0.27	16	0.22	0.08	0.1269
**B2M (Final)**	8	0.56	0.51	14	0.21	0.12	0.3894
**CyC (Basal)**	7	1.75	0.93	4	0.76	0.17	0.035 *
**CyC (Final)**	8	1.71	1.12	2	0.8	0.14	0.3054

**Table 4:** **Summary of correlations between plasma biomarkers and gene expression of *NOS3*, *VEGFA*, and *TNF*.** * Indicates that this condition was also found significantly associated in non-DM population of the GHAS trial.

	Plasma Marker/ Gene	*NOS3* (Basal)			*NOS3* (Final)	*VEGFA* (Basal)		*VEGFA* (Final)	*TNF* (Basal)	
		*Both*	*Placebo*	*GH*	*GH*	*Both*	*GH*	*Placebo*	*Both*	*GH*
	**TNF-α (Basal)**	r = −0.49 *p* = 0.015		r = −0.78 *p* = 0.0064		r = −0.432 *p* = 0.039	r = −0.773 *p* = 0.005		r = 0.588 *p* = 0.035 *	
	**TNF-α (Final)**		r = 0.866 *p* = 0.012							
	**TNF-α > 8.1 (Basal)**								r = 0.802, *p* = 0.001 *	
	**hsCRP (Basal)**			r = 0.74 *p* = 0.009		r = −0.632 *p* = 0.005		r = −0.775 *p* = 0.041		
	**hsCRP > 0.5 (Basal)**			r = −0.693 *p* = 0.018		r = −0.546 *p* = 0.019		r = −0.866 *p* = 0.012		
DM	**hsCRP > 0.5 (Final)**				r = −0.711 *p* = 0.021 *			r = −0.866, *p* = 0.012		
	**NLR > 3 (Basal)**			r = −0.645 *p* = 0.032						
	**NLR > 5 (Basal)**			r = −0.69 *p* = 0.018						
	**HbA1C (Basal)**									r = 0.728 *p* = 0.026
	**HbA1C (Final)**							r = −0.975 *p* = 0.005		
Non-DM	**hsCRP (Basal)**				r = −0.9 *p* = 0.037					
	**LDLc**					r = −0.827 *p* = 0.002				

**Table 5:** **Summary of correlations between plasma biomarkers and gene expression of *IGF-1*, *MSTN*, *MYOG*, *KDR*, and *NOX4***. * Indicates that this condition was also found significantly associated in non-DM population of the GHAS trial.

	Plasma Marker/ Gene	*IGF-I* (Basal)		*IGF-I* (Final)	*MSTN* (Basal)	*MSTN* (Final)	*MYOG* (Final)		*KDR* (Final)	*NOX4* (Basal)	*NOX4* (Final)
		Both	Placebo	GH	Both	GH	GH	Placebo	Placebo	Both	*GH*
	**TNF-α (Basal)**	r = 0.519 *p* = 0.011	r = 0.821 *p* = 0.0341					r = 0.8 *p* = 0.023 *		r = 0.305 *p* = 0.037	
	**TNF-α > 8.1 (Basal)**	r = 0.598 *p* = 0.003			r = 0.586 *p* = 0.011						
	**hsCRP (Basal)**			r = −0.648 *p* = 0.031							
	**hsCRP (Final)**					r = −0.691 *p* = 0.027					r = 0.709 *p* = 0.003 *
DM	**hsCRP > 0.5 (Final)**										r = 0.773 *p* = 0.001 *
	**NLR > 3 (Basal)**			r = −0.662 *p* = 0.019							
	**NLR > 5 (Basal)**								r = −0.857 *p* = 0.014 *		
	**HbA1C (Basal)**	r = 0.597 *p* = 0.019									
Non-DM	**TNF-α (Basal)**									r = 0.645 *p* = 0.032	
	**TNF-α > 8.1 (Basal)**									r = 0.717 *p* = 0.009	
	**hsCRP (Basal)**						r = 0.9 *p* = 0.037		r = −0.847 *p* = 0.016		

**Table 6:** **Mortality in the GHAS trial**. Short-term mortality: 0–2 months (period of treatment). Long-term mortality: 2–12 months (observation period).

	0–2 Months	Mortality	*p*-Value	2–12 Months	Mortality	*p*-Value
**Placebo**	0/16	0%		2/16	12.5%	
**GH**	1/18	5.5%	0.42	5/17	29.4%	0.23
**Cumulative**		5.5%			47.4%	

**Table 7:** **Plasma and clinical predictors of mortality in the GHAS trial.** COPD: Chronic Obstructive Pulmonary Disease; ASA: The American Society of Anesthesiologists (ASA) class is a system for assessing the risk of patients before surgery. ASA 3 and 4 include patients in poor (ASA 3) and extremely poor (ASA 4) physical condition for surgery. CI: Confidence Interval. (*p* < 0.05 is considered to be significant).

Predictors of Mortality in the GHAS Trial			
	*p*-Value	OR	CI (95%)
**NLR ≥ 3 (Basal)**	0.019	6.9	0.71–353.7
**TNF-α ≥ 8.1 (Basal)**	0.0487	2.5	0.23–136.6
**COPD**	0.042	5.8	0.84–40.7
**ASA4-ASA3**	0.0119	15.7	0.87–284.9

## Data Availability

The data presented in this study are available on request from the corresponding author. The data are not publicly available due to data protection policy in Galicia, Spain.
